# Nanotechnology-Based Medical Devices for the Treatment of Chronic Skin Lesions: From Research to the Clinic

**DOI:** 10.3390/pharmaceutics12090815

**Published:** 2020-08-27

**Authors:** Marco Ruggeri, Eleonora Bianchi, Silvia Rossi, Barbara Vigani, Maria Cristina Bonferoni, Carla Caramella, Giuseppina Sandri, Franca Ferrari

**Affiliations:** Department of Drug Sciences, University of Pavia, Viale Taramelli 12, 27100 Pavia, Italy; marco.ruggeri02@universitadipavia.it (M.R.); eleonora.bianchi04@universitadipavia.it (E.B.); silvia.rossi@unipv.it (S.R.); barbara.vigani@unipv.it (B.V.); cbonferoni@unipv.it (M.C.B.); carla.caramella@unipv.it (C.C.); franca.ferrari@unipv.it (F.F.)

**Keywords:** chronic wounds, nanotechnologies, medical devices, critical quality attributes, biocompatibility, wound models, clinical evaluation, CE marking

## Abstract

Chronic wounds, such as pressure ulcers, diabetic ulcers, venous ulcers and arterial insufficiency ulcers, are lesions that fail to proceed through the normal healing process within a period of 12 weeks. The treatment of skin chronic wounds still represents a great challenge. Wound medical devices (MDs) range from conventional and advanced dressings, up to skin grafts, but none of these are generally recognized as a gold standard. Based on recent developments, this paper reviews nanotechnology-based medical devices intended as skin substitutes. In particular, nanofibrous scaffolds are promising platforms for wound healing, especially due to their similarity to the extracellular matrix (ECM) and their capability to promote cell adhesion and proliferation, and to restore skin integrity, when grafted into the wound site. Nanotechnology-based scaffolds are emphasized here. The discussion will be focused on the definition of critical quality attributes (chemical and physical characterization, stability, particle size, surface properties, release of nanoparticles from MDs, sterility and apyrogenicity), the preclinical evaluation (biocompatibility testing, alternative *in vitro* tests for irritation and sensitization, wound healing test and animal wound models), the clinical evaluation and the CE (European Conformity) marking of nanotechnology-based MDs.

## 1. Introduction

A wound can be defined as damage to or an interruption of the normal anatomical structure of the skin, following a physical or thermal trauma or as a consequence of underlying medical or pathophysiological conditions. It can result in a simple injury of the skin epithelial integrity or it can be deeper and involve the subcutaneous tissue, extending to tendons, muscles, vessels, nerves, and bones [1,2].

The wound healing is a very complex and dynamic process that occurs in five subsequent or overlapped phases (hemostasis, inflammation, migration, proliferation and remodeling), aiming to restore the injured tissue integrity and functionality. It involves a complex series of events including chemotaxis, cell division, neovascularization, synthesis of a new extracellular matrix, and the formation and remodeling of scar tissue. These events require the intervention of various mediators including platelets, inflammatory cells, cytokines, and growth factors [3,4]. The wound healing process is generally influenced by several factors that can be divided into intrinsic (ischemia, infections, presence of necrotic tissue) and extrinsic (diabetes, cancer, chronic diseases, steroid therapy, radiation and malnutrition) and the combination of these factors is crucial for the repair of injuries [5].

An orderly and timely healing path, to obtain functional and anatomical recovery, characterizes acute wounds and the healing time usually varies from 5 to 10 days, up to 30 days. Considering this, chronic wounds are commonly defined as lesions that fail to proceed through the normal healing process to restore skin anatomical and functional integrity within a period of 12 weeks. The treatment of chronic skin lesions still represents a great challenge. This problem particularly affects the most developed countries, which have a higher incidence of metabolic pathologies that often predispose to unhealed wounds. Chronic wounds are classified into four categories: pressure ulcers, diabetic ulcers, venous ulcers and arterial insufficiency ulcers [6]. The prevalence of ulcers ranges from 1% in the adult population to 3–5% in the population over 65 years of age with high treatment costs. Chronic wound care generally costs 2–3% of total healthcare budgets [7]. Moreover, since the treatment of chronic injuries is expensive and complex, its failure is particularly demanding and could lead to extremely serious consequences such as the onset of persistent infections, eventual limb amputation and ultimately death. Considering the ageing of the EU population and the increase of related pathologies, challenges, such as the strengthening of healthcare systems, the continuous enhancement of quality of life, and the active ageing, are to be faced. In particular, chronic wounds have a negative impact on patients and society since patients can experience pain, significant emotional and physical distress, reduced mobility and social isolation and, consequently, emotional and physical trauma involving also patients’ families. Currently no ideal treatment has been identified, and chronic wounds might require several years to heal, and some remain unhealed for decades. Amputations remain the last option after all therapeutic interventions have been exhausted resulting in disabilities. In fact, ulcers precede 85% of amputations and diabetic ulcers are the reason for 70% of all lower limb amputations. In diabetic patients 5-year mortality rate following amputation ranges from 40 to 70% [8,9,10].

Although the prevalence is similar to that for heart failure, the morbidity and associated costs of chronic wounds, including amputation and death, have been largely ignored, since wounds are typically managed as a comorbidity of other conditions, limiting the impact of efforts to overcome the growing challenge they represent. Clinicians often lack specific training for wound diagnosis and its treatment because different specialists, such as dermatologists, surgeons, endocrinologists, podiatrists, vascular surgeons, and geriatricians, are involved depending on the comorbidity affecting the patient. This fragmented responsibility has led to a difficult appraisal of the prevalence related to each lesion type and the wound management costs and there is no univocal approach among the various countries. In addition, the lack of prioritization of resources and capabilities around wound care cause prolonged and inconsistent treatments and an uncoordinated approach to prevention. Non-healing and infected wounds are actually a silent epidemic, thus resulting in inadequate planning and poor implementation of prevention, treatment and management strategies. So far wound healing could be considered as a neglected disease [11,12].

Although many options are available to treat these types of lesions from conventional and advanced dressings, implants, up to skin grafts, none of these are generally recognized as a gold standard. In this context, the need for effective therapies, capable of reducing the time of wound closure and to consequently improve the quality of life of the patients, emerges. The characteristics of a lesion (as size, type, and depth) lead to the product choice. However, these are often ineffective and innovative approaches are in development. Among the various advanced systems, medical devices (MDs) based on nanotechnologies have aroused interest, although, currently, there are only a few products in the pharmaceutical market. These boast considerable advantages over the use of conventional medications, representing concrete options to efficiently manage non healing wounds, primarily generating health benefits for the patients and secondly economic advantages for healthcare systems.

Based on the recent developments, this paper reviews nanotechnology-based MDs intended as skin substitutes. In particular, nanofibrous scaffolds are here emphasized. The discussion will be focused on the definition of critical quality attributes, MDs preclinical and clinical evaluation and the CE marking.

## 2. Medical Devices: Definition and Classification

MDs have a crucial role in healthcare: they are fundamental in the diagnosis, prevention, monitoring and treatment of diseases and in improving the quality of life of patients. The Legislative Decree No. 46 of 24 February 1997 of WHO (World Health Organization) defines the MDs as: “any instrument, apparatus, implement, machine, appliance, implant, reagent for *in vitro* use, software, material or other similar or related article, intended by the manufacturer to be used, alone or in combination, for human beings, for one or more of the specific medical purpose(s) of:

− diagnosis, prevention, monitoring, treatment, or alleviation of disease,
− diagnosis, monitoring, treatment, alleviation of or compensation for an injury,
− investigation, replacement, modification, or support of the anatomy or of a physiological process,
− supporting or sustaining life,
− control of conception,
− disinfection of MDs,
− providing information by means of *in vitro* examination of specimens derived from the human body.

A MD does not achieve its primary intended action by pharmacological, immunological or metabolic means, in or on the human body, but which may be assisted in its intended function by such means” [13].

The MDs market represents a third of the global market, generating a turnover of approximately USD 423.8 billion (2018 estimate) of annual revenues: this reveals the socio–economic impact of the MD sector in the world [14].

MD classification is a fundamental step in the design and the development of the device, since the class assigned determines the procedure to assess its conformity for CE marking and its entrance into the market [15]. This classification is based on the level of risk associated to the MD in development. In particular, Annex IX of the Directive 93/42/EEC (European Economic Community) classifies the MDs in four classes of increasing risk (Table 1):

− class I: less critical (low risk) devices, such as most of the non-active and non-invasive ones (two subclasses can be identified within class I: sterile class Is—those supplied in a sterile state—and class Im—those that perform a measurement function);
− class IIa: medium risk devices, such as some non-active devices (invasive and non-invasive) and active devices that interact with the body in a non-dangerous way;
− class IIb: medium/high risk devices, such as some non-active devices (invasive species) and active devices that interact with the body in a dangerous way;
− class III: high-risk devices, such as most of those implantable, those containing drugs or animal derivatives and some MDs that act on the functions of vital organs.

The conformity assessment procedure should be appropriate to the MD class: in the cases of medium–high risk MDs, this process involves a Notified Body to certify the procedures. The essential requirements are mandatory, but each manufacturer may decide the technical standards to apply among international (ISO), European (EN), national (UNI) ones [15].

The Directive 93/42/EEC defines the MD classes on different parameters such as the degree of invasiveness, the vulnerability of the patient considered, the contact duration, the potential risks associated with the intended use and the MDs intrinsic characteristics, and its dependence on an energy source (active device) [16]. In particular, the duration could be temporary (less than 60 min), short-term (less than thirty days), and long-term (over thirty days) and the degree of invasiveness is related to the penetration either into any part of the body through an orifice, or into the skin [17]. Surgical MDs could temporarily penetrate through the surface of the body, both in a surgical intervention (such as a scalpel) and outside of this context (syringe needle) or could be permanently implantable (to replace an epithelial surface). As for the active devices the energy could be generated directly by the human body or by an external source [18].

For class I devices (typically non-active and non-invasive), the manufacturer is responsible for the evaluation procedure, since the risk associated to the MDs is low. However, if class I devices have a measuring function (Im) or are sterile (Is), a Notified Body should verify critical aspects, such as the measuring function and the sterilization process. For class IIa, IIb and III devices, the involvement of the Notified Body is mandatory proportionally to the risk class: in particular class III devices (including implantable devices and devices containing drugs) require MDs design approval and the manufacturing process must take place in a quality management system before marketing [17,18].

## 3. Medical Devices for Wound Healing

Wound healing MDs could belong to all the classes depending on the technology and intended use. Considering the application, MDs should be selected considering the wound stage and aim to repair damaged tissue and to restore skin function. These range from the relatively simple devices, such as paraffin gauze, to complex artificial skins and scaffolds that may or may not contain bioactive factors to promote angiogenesis or reepithelialization [19,20]. In particular, the FDA (Food and Drug Administration) has determined that wound devices intended to promote the healing process and which are used as grafting to replace full-thickness lesions, are classified as class III MDs [21]. Both three-dimensional scaffolds and wound dressings are able to cover and to protect wounds against biofilm attachment, but only three-dimensional (3D) scaffolds promote cell proliferation by resembling the extracellular matrix and by replacing the damaged tissue.

### 3.1. Current Commercially Available Skin Substitutes in Wound Healing

Several bioengineered skin substitutes, such as living skin constructs and acellular matrices, have been developed [20].

Living skin constructs use transplanted cells, such as fibroblasts and keratinocytes, embedded into matrices that should promote the healing process. Apligraf^®^ was the first bioengineered living cell therapy approved by the FDA, in 1998 for the treatment of venous ulcers, and in 2000 for the treatment of diabetic foot ulcers, as a class III MD [22]. Apligraf^®^ is a by-layered skin substitute: the epidermal layer is based on neonatal keratinocytes and the dermal layer is based on neonatal fibroblasts seeded into a bovine type I collagen matrix [23]. The cells of these bioengineered skin substitutes are capable of producing a large number of cytokines and growth factors, which play a fundamental role in the wound healing process [20,24]. Dermagraft^®^ is a class III MD approved by the FDA in 2001 to treat diabetic foot ulcers. It is based on a bioresorbable polyglactin mesh scaffold with integrated fibroblasts [25]. In this structure, fibroblasts proliferate across the mesh and secrete collagens, and growth factors. [26,27]. Another living skin substitute is TheraSkin^®^, a human skin allograft obtained from cadavers within 24 h after death. The tissue is cleaned, treated with antibiotics and cryopreserved to protect viable cells and extracellular components. However, unlike Apligraf^®^ and DermaGraft^®^ classified as MDs, TheraSkin^®^ is a human allograft, and therefore subject to the transplant regulations [28].

Acellular matrices may be synthetic or based on physiological components or may be derived from animals or humans, and in these cases the cells are removed during the production process, to reduce inflammatory or immune responses [20]. Acellular matrices are generally classified as implantable class III MDs and are mainly based on collagen, such as Oasis^®^, Integra™ and Promogran™.

The Oasis^®^ Wound Matrix is an extracellular matrix (ECM) derived from the submucosal layers of the pig intestine. It is composed by collagen (mainly type I collagen), elastin, glycosaminoglycans, proteoglycans, and growth factors, and is approved as a class II MD [29,30]. Integra^®^ Dermal Regeneration Template is a MD based on a two-layer skin regeneration system: the inner layer (dermal) is made of an acellular 3D matrix of bovine collagen rich in chondroitin sulphate, while the outer layer (epidermal) is made of a thin silicone sheet that simulates epidermis and avoids fluid loss [20,22]. Promogran™ is a sterile, freeze-dried dressing made of natural materials (55% collagen and 45% oxidized regenerated cellulose), it is resorbable after the implant [31]. Promogran™ Prisma is a version of Promogran™ that includes silver ions with antimicrobial properties [32].

Other commercial acellular matrices are Hyalomatrix^®^ and Talymed^®^, able to stimulate ECM synthesis to restore skin architecture.

Hyalomatrix^®^ is a sterile and flexible wound scaffold consisting of a double layer: an internal layer, which promotes dermal reconstitution based on Hyaff (benzyl ester of hyaluronic acid) and a surface layer consisting of a semi-permeable silicone membrane. Hyaff, which is biodegradable, when in contact with the wound, acts as a three-dimensional scaffold for cellular invasion and neoangiogenesis, while the silicone layer controls the loss of water vapor and provides wound protection [33,34]. Talymed^®^ is composed of nanofibers of poly-*N*-acetyl glucosamine, isolated from microalgae, and indicated for the treatment of several complex wounds. This is able to promote wound healing mainly by re-epithelialisation, stimulation of angiogenesis, and cell proliferation [35,36].

The health expenditure related to chronic wound healing is high. Carter et al. determined the cost-effectiveness of three cell/tissue derived products in relation to standard care in the treatment of venous leg ulcers. In particular, this study evaluated Oasis^®^, Apligraf^®^ and Dermagraft^®^. A three-state Markov simulation model (unhealed, healed, and dead) was used weekly for 1 year and the final model outputs were cumulative costs, clinical outcomes, and the incremental cost-effectiveness ratio at 1 year. The Oasis^®^ cost-effectiveness was dominant compared to Apligraf^®^ and Dermagraft^®^: in particular, the expected costs for Oasis^®^, Apligraf^®^ and Dermagraft^®^ in venous leg ulcers were USD 6732, USD 10,638 and USD 11,237, while the expected results were 31, 29 and 27 ulcer-free weeks, respectively, showing clear cost advantages in the use of the ECM-based acellular matrix [37].

### 3.2. Nanotechnology-Based Medical Devices for Wound Healing

In recent years, research has focused on the development of bioengineered skin substitutes, which simulate multiple characteristics of the native ECM [38].

Nanotechnologies represent an interesting field with multiple promising applications in medical practice and the interest is currently transversal to various scientific areas for multiple reasons. In fact, nanotechnology-based MDs possess superior surface-to-volume ratios and are consequently capable of deep interaction with the biological target at the wound bed [39,40,41,42].

In particular, nanofibrous materials have been extensively explored as scaffolds for skin regeneration, since they could better resemble the ultrastructure of the ECM. In fact, scaffolds are not passive mechanical supports, but actively participate in the regeneration of the tissues, providing a 3D structure for the cell homing and the tissue formation [39,40,41]. Scaffolds are constructs capable of recruiting native cells and of stimulating tissue reparation by promoting the initial cell adhesion, proliferation and differentiation, and supporting native ECM formation. In fact, the scaffold ability to mimic the tissue structure and its properties seems to be fundamental for the regeneration process [41,43]. An ideal scaffold for skin regeneration should be able to satisfy peculiar characteristics and properties. After their grafting, the scaffolds should ideally be resorbed by degradation upon newly tissue formation with negligible immune reaction to avoid the onset of a serious inflammatory response [38,39,40,41].

Natural polymers are commonly used in the development of nanofibrous scaffolds for skin tissue engineering due to their biocompatibility and their ability to simulate the ECM structural, biomechanical, and biochemical functions. The major advantage of these polymers is their versatility (e.g., hydrophobicity, charge, size) [44,45]. These materials should be easily sterilizable and have acceptable levels of toxicity. The most common natural or naturally derived polymers used in scaffold development are collagen [46,47,48], gelatin [49,50,51], chitosan [52,53,54,55] and alginate [56].

Synthetic polymers such as PCL (polycaprolactone), PLA (polylactic acid), and PLGA (polylactic-co-glycolic acid) have been also proposed for the development of nanofibrous scaffolds with wound healing applications [57,58,59,60].

**Figure 1:**
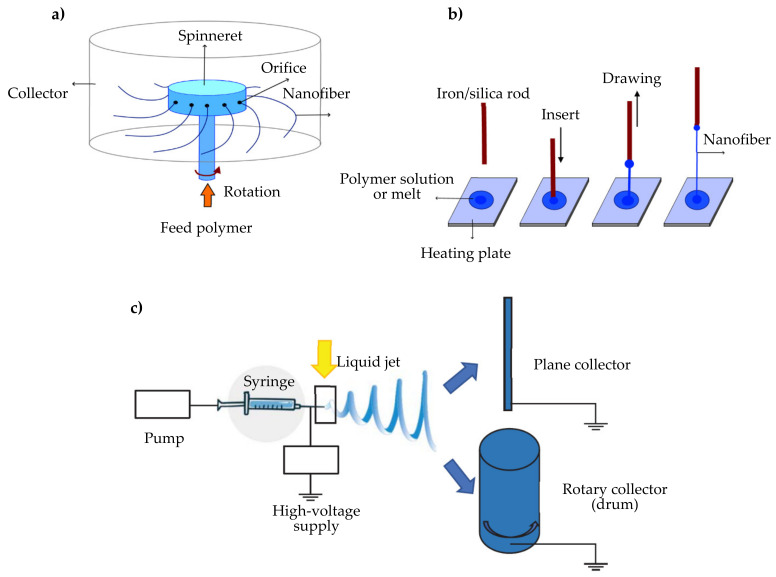
(**a**) Centrifugal spinning, (**b**) direct drawing technique and (**c**) electrospinning apparatus. Modified from [61] with permission.

Different techniques are used for the manufacture of nanofibers such as centrifugal spinning, the direct drawing technique, and electrospinning [61] (Figure 1). In particular, electrospinning is a simple and versatile technique used in continuous manufacturing, extremely pushed by regulatory agencies to improve product quality; moreover it is considered an advanced, easily scalable, and emerging technology, leading to improvements in terms of new product properties, production speed, cost, and waste and pollution management [62].

Moreover, hemoderivatives and metallic nanoparticles have been successfully incorporated into nanofibrous scaffold to generate innovative constructs able to further promote the healing process [63,64]. In fact, hemoderivatives such as growth factors play a pivotal role in the regulation of the healing process, promoting cell proliferation and metabolism through interaction with specific membrane receptors and inducing cell migration, as leukocytes and fibroblasts, in the wound bed. The main obstacles to growth factor delivery are the poor stability and the short retention times at the application site. The association of growth factors in a 3D solid scaffold should allow the attainment of a controlled release, providing protection from degradation and keeping them for longer periods in contact with the wound bed [65].

**Figure 2:**
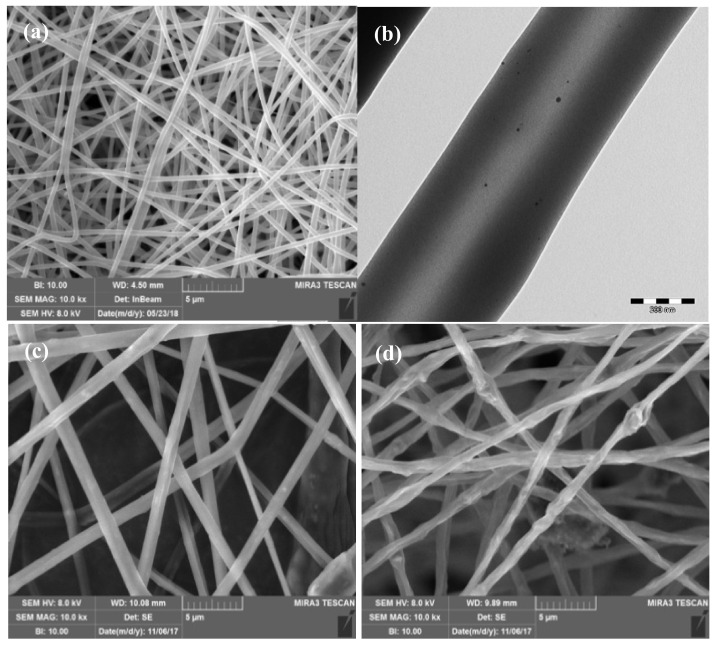
Images of nanofibrous scaffolds as dermal substitutes: (**a**) polymer-based matrix; (**b**) polymer-based matrix embedded with silver nanoparticles; (**c**) polymer-based matrix embedded with halloysite and (**d**) with montmorillonite adapted from [53,54,55]. CC BY 4.0.

Metallic nanoparticle loaded scaffolds possess peculiar properties such as low toxicity and antibacterial activity, making them promising candidates for the treatment of infected wounds. Thanks to their peculiar properties, nanoparticles (i.e., silver [53,66,67,68], copper [69] and zinc oxide nanoparticles [70,71]) seem a valid option to control bacterial infections, overcoming antibiotic-resistance mechanisms owing to their unique and advantageous physio-chemical properties. In fact, the interaction of metallic nanoparticles and microorganisms causes the production of reactive oxygen species (ROS), damaging bacterial cells. Moreover, metallic nanoparticles possess the ability to bind DNA or RNA, and could be advantageous for biofilm eradication [72,73]. These nanoparticles could either be loaded into the polymeric matrix or could be absorbed and coated onto a MD surface: this loading should confer antimicrobial properties to these constructs with an increasing effect over time. Figure 2 reports the images of nanofibrous scaffolds as dermal substitutes: (a) polymer-based matrix [55]; (b) polymer-based matrix embedded with silver nanoparticles [53]; (c) polymer-based matrix embedded with halloysite [54] and (d) with montmorillonite [54].

## 4. Quality by Design (QbD) Approach

The translation from research to clinic of nanosystem-based MDs is deeply related to the product quality and the product performance. The development of a new product from the idea to the market can be defined by different steps, described using the technology readiness level (TRL) from 1—idea to 10—market that estimates the maturity of the technologies. Considering this, thorough understanding of product and process, the QbD approach allows for better supporting product specifications and control of the manufacturing process. The ICH (International Conference on Harmonisation) guideline Q8R2 entitled “The quality target product profile” forms the basis for the design and the development of the product: this describes the final expectation of the final product (Table 2).

**Figure 3:**
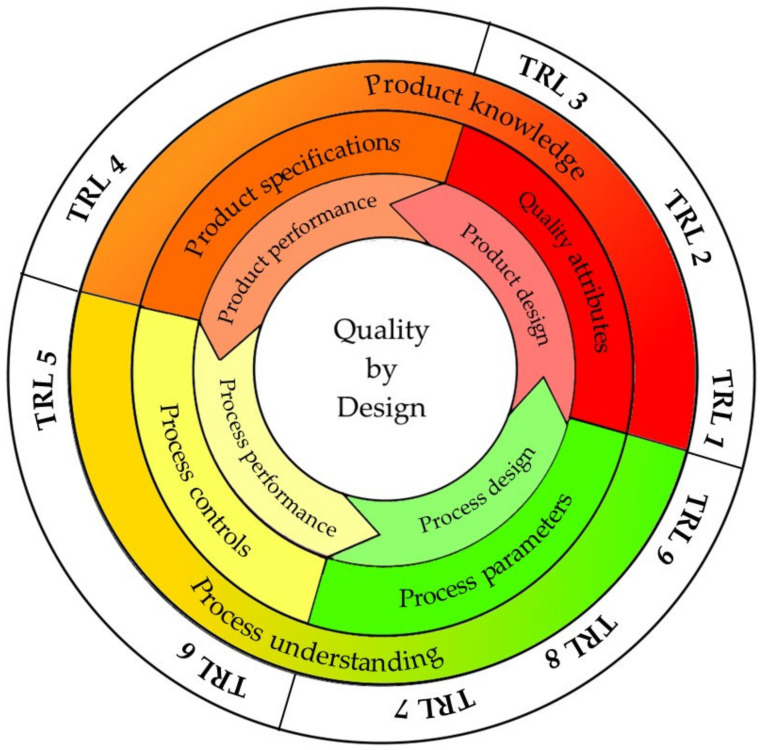
Concept of Quality by Design (QbD) for the development of medical devices (MDs) and the different steps with the corresponding technology readiness level (TRL). Modified from [74]. CC BY 4.0.

Figure 3 describes the concept of QbD for the development of MD and the different steps with the corresponding TRL.

In particular, the characteristics of nanotechnology-based MDs significantly depend on the manufacturing parameters. A considerable number of operating parameters and their variables often interfere in the manufacturing process. Furthermore, it is not always possible to establish a precise relationship between the technological process specifications and the properties of the resulting product. For this reason, regulatory agencies require a high characterization degree of the finished product, the production phases and the equipment used [75].

Therefore, it emerges how relevant are the identification and the monitoring of critical process parameters (CPP) and critical quality attributes (CQA), in the context of good manufacturing practice (GMP). According to ICH Q8R2, the CPPs are defined as the set of operating parameters capable of significantly influencing the CQAs. CPPs identify a predefined acceptance interval of the process parameters to achieve the desired product, while CQAs are the product attributes influencing safety and quality [75]. Safety assessments should consider that nanosystem properties could vary in the research and development phase, before the scale up and manufacturing phases. CQA knowledge and analysis are the keys to obtain a safe product, minimizing the use of useless tests and preventing unsafe output. Moreover, the product quality and CQA are better defined considering the quality risk management according to ICH Q9.

All these tools allow the setting up of the product and process safely by design to reduce the MD risk and facilitate the clinical testing. Moreover, other obstacles on the path from research to clinic are associated with the scale up of the nanosystems. In fact, in the first phases of research and development, nanosystems are generally produced in small quantities (micrograms or milligrams), with techniques difficult to scale up: this could have a negative impact on the subsequent phase of the development process, considering the strict dependence of the nanoproduct characteristics on the process variables. The small scale of production renders the quality assessment for MD registration difficult since quality control requires significantly high volumes of materials. Moreover, the process complexity could negatively interfere with the CPPs identification and their reproducibility [76].

**Figure 4:**
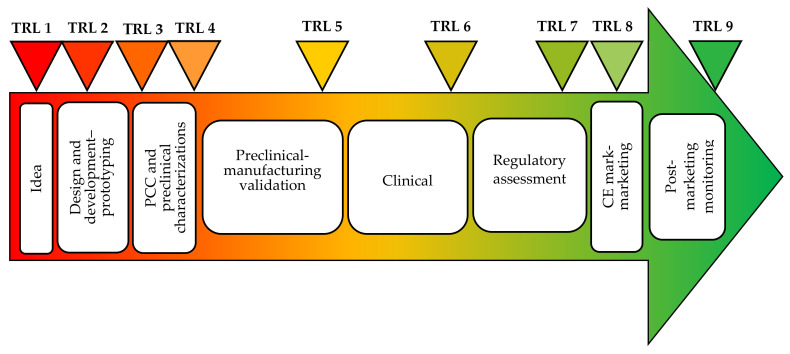
From the idea to the market of a MD. Modified from [77]. CC BY 4.0.

Physicochemical characterization (PCC) and preclinical characterization are fundamental to assessing MD quality, safety, and efficacy, and are the pillars which describe the product. Figure 4 reports the development stages from the idea to the market of a MD with the different phases and the corresponding TRL.

## 5. Definition of the Quality Attributes

The definition of quality attributes requires multiple tests, and ISO guidelines are generally adopted although they are not specifically developed for nano based systems. However, modified procedures could be considered to be adapted to the scope. Table 3 reports a summary of Quality Attributes (QA) for characterization of MDs, their descriptions, the methods used and the bibliographic references.

### 5.1. Chemical and Solid State Assessement

ISO 10993 described the chemical assessment, such as chemical characterization of materials (ISO 10993-12, 18, 19), establishment of allowable limits for leachable substances (ISO 10993-17), identification and quantification of degradation products (ISO 10993-13, 14, 15), determination of tolerable intake for extractable substances (ISO 10993-17), ethylene oxide sterilization residuals (if applicable) (ISO 10993-7).

MDs chemical characterization focuses on the evaluation of the types and the quantities of chemicals that may migrate from the device in both typical usage (leachables) and when challenged (extractables). If the MD is based on polymeric materials, chemical evaluation should include the bulk polymer characterization, as a starting material (ISO 10993-13, Section 5.2): this is crucial in the case of natural polymers, particularly susceptible to contamination of allergens in traces, such as chitin and chitosan (extracted from crustaceans, [78]), or animal-derived pathogens, as collagen (from mammals). Moreover, the surface composition and reactivity are generally characterized by X-ray photoelectron (XPS) spectroscopy.

Many chromatographic/analytical techniques (UV-vis, HPLC, GC/LC-MS) should be considered depending on the type of materials. For example, collagen should be examined by means of chromatography to determine its content in hydroxyproline, tyrosine, tryptophan and cysteine [12], while for synthetic or semi-synthetic materials, the analysis of the exact molecular structure is recommended [79].

A complete solid-state analysis is essential for quality and safety controls. The possible presence of different polymorphic forms is associated with different melting points and with different dissolution rates. For example, silk fibroin, widely used in wound care, can be obtained with different degrees of crystallinity depending on the process conditions [80], while cellulose and chitin could be characterized by six and three polymorphic forms, respectively [78,81].

In addition to the crystal structure, the solid morphology influences its processing procedures. Solid-state characterization requires thermal analysis (TGA/DSC), X-ray diffractometry or NMR spectroscopy [78].

### 5.2. Stability

The MD stability is a fundamental requirement. In the case of nanotechnology-based MDs this concept assumes a broader relevance and it concerns various aspects, such as chemical and physical stability, and in particular tendency for aggregation, growth of crystals, variation of solubility and dissolution, solid state morphology, as well as the adsorption of components of the formulation should be carefully evaluated [82]. Currently, no specific assessment is recommended and ICH Q1A (R2), Q1C (R2) and Q5C guidelines are normally extended to this area [83,84,85]. ICH Q1A refers to new drugs, Q1C to new pharmaceutical forms of approved drugs and Q5C to formulations containing biological and biotechnological drugs. These describe the selection criteria considering the batch quantity (generally at least three) and suggest the evaluation of product batches, manufactured from different batches of raw materials, as well as the conditions and times prescribed. The objectives of these tests are the identification of the degradation pathways to which the system is susceptible, and of an expiry date considering the environmental parameters (temperature and humidity) and the duration time; this should allow the manufacturer to identify the storage conditions (room temperature, could storage or frozen) to ensure product stability [82]. The stability tests could be performed in normal conditions (short, intermediate and long term) and in accelerated conditions accordingly to the product criticality [83]. Moreover, if a component is photosensitive, a photostability assessment, as recommended in ICH Q1B, is particularly important [86].

If the system contains peptidic/proteic components, special storage conditions could be often required according to ICH Q5C.

### 5.3. Particle Size

Considering the nanotechnology-based MDs, the particle size is a key parameter in nanoscale products and determines their properties. In fact, the particle size regulates the interaction with the biological systems and the formation of the protein corona, as well as the biodistribution for passive targeting. The particle size could also influence the kinetics of biodegradable polymers [87], and thermal behavior compared to the single components [88].

Considering that a single data point (average particle size) cannot adequately describe a distribution of points (size distribution), it is suggested to include other size parameters in order to describe size distribution. D10, D50, D90 diameters are commonly used to describe particle size distribution and correspond to the volume diameters at 10, 50 and 90% of cumulative volume distribution, along these the span of a volume-based size distribution (SPAN = (D90−D10)/D50)) is also considered and this is an index to indicate how far the D10 and D90 diameters are apart, normalized with the midpoint (D50).

FDA recommends the reporting of the average diameter (d10, d50, d90) and the polydispersion index to characterize the size distribution. This is important to study the tendency to aggregate. Aggregate formation by Van der Waals interaction determines the potential loss of the product properties; therefore, regulatory authorities require the evaluation of particle size by comparing at least two methods [89]. Generally, it is necessary to resort to multiple complementary techniques, always including an investigation by electron microscopies (scanning electron microscopy—SEM, or transmission electron microscopy—TEM) [79,90] although these are not free from critical issues. SEM analysis requires sample sputtering with graphite or gold/platinum and the sample exposure to an electron beam in vacuum conditions: these could damage the system morphology [87,91]. While TEM allows a higher resolution: but this requires longer and more complex sample preparation compared to SEM [87].

To date, there is no single method that can cover the entire dimensional range. Mainly regulators recommend the use of SEM, TEM, X-ray diffractometry, DLS (Dynamic light scattering), AFM (atomic force microscopy) and NTA (nanoparticle tracking analysis).

Tunable resistive pulse sensing (TRPS) is an innovative technique that uses the Coulter principle. Each particle is forced to pass through a calibrated nanopore with a tunable diameter causing a current pulse proportional to its volume. In particular, the reduction of current peak depends on the particle size and it is possible to trace the particle concentration by the frequency of these, as well as the zeta potential [92].

AFM and SEM are also able to assess the system morphology, which is extremely challenging in implantable MDs.

### 5.4. Mechanical Properties

Native skin shows tensile strength values between 5.0 and 30.0 MPa, a Young’s modulus in the range of 4.6–20.0 MPa and elongation at break of about 35.0–115.0%; these values are wide since the skin mechanical properties vary depending on the body area, and the ageing (thinner and less flexible [55,56,57,58,59,60,61,62,63,64,65,66,67,68,69,70,71,72,73,74,75,76,77,78,79,80,81,82,83,84,85,86,87,88,89,90,91,92,93]). The ability of the scaffold to maintain its integrity during grafting is related to the tensile strength, while the performance of the scaffold upon grafting is related to elongation and the Young’s modulus. Furthermore, scaffolds intended as dermal substitutes should exhibit good mechanical strength to ensure fibroblast adhesion and proliferation [93].

For these reasons, mechanical properties are essential factors for the success of implants.

Compressive, tensile and flexural tests are the most common techniques to assess scaffold performance. However, while a scaffold applied to bone requires a compressive test, the tensile or flexural tests are more important to evaluate scaffolds intended as skin or cartilage substitutes [93].

The tensile test, defined by the ISO 6892-1:2019 (Metallic materials—Tensile testing—Part 1: Method of test at room temperature), is the most important among the destructive mechanical tests. In this test, two equal and opposite loads are applied to the edges of the sample, so that inside the structure there is a direct distribution of forces along the application axis of the loads. In this way, the structure stretches in the axial direction and tapers along the other two directions [94].

In the flexural test, the loads are applied so that the structure undergoes a curvature along its axis, and it is subjected to both tension and compression stresses (ISO 178:2019 Plastics—Determination of flexural properties). In this test, it is possible to realize flexural stresses at several points, mimicking the *in vivo* application [95].

### 5.5. Surface Properties

In addition to XPS and SEM and AFM to assess surface composition and reactivity, and system morphology, respectively, the zeta potential is the most relevant CQA for the characterization of nanoproducts and it is the most common criterion to describe the colloid surface charge. Each particle has its own surface charge, and this generates a counter ions layer, the Stern layer, around it. This has ionic interactions with the nanoparticle and follows it in its motion within the medium. More externally, both negatively and positively charged ions form a double layer and a slipping plane faces the solvent bulk. The zeta potential is the electrical potential at the slipping plane [96]. For zeta potential values ranging from ±10 mV, the particle is considered almost neutral and exposed to the risk of aggregation, while for values out of ±30 mV range, the particles are considered strongly cationic/anionic. The determination of the zeta potential depends on the medium pH and its ionic strength. The most common technique for determining the zeta potential is LDV-PALS (laser Doppler velocimetry-phase analysis light scattering), which measures the Doppler effect induced by the movements of the particles within an electric field. Electrophoretic mobility is related to the zeta potential through Henry’s equation, which takes into account the dielectric constant and the medium viscosity. LDV-PALS results in an average value of the zeta potential, more representative for monodisperse systems than for polydispersed ones [87].

However, the surface zeta potential of a complex three-dimensional (3D) structure with a high aspect ratio (i.e., a dimension in the nanometric range and the others in micron- or milli-meter ranges) cannot be measured by means of the electrophoretic mobility.

In these cases, the evaluation of the streaming potential and streaming current allows the assessment of the surface zeta potential and surface swelling; the fundamental equation that relates the streaming potential and the streaming current to the zeta potential has been derived by Hermann von Helmholtz and Marjan von Smoluchowski:

$$\zeta =\frac{dl_{str}}{d\Delta p}\times \frac{\eta }{\varepsilon \times \varepsilon_{0}}\times \frac{L}{A}$$

where *dl/dp* is the slope of streaming current vs differential pressure, *η* the electrolyte viscosity, *ε* the dielectric coefficient of electrolyte, *ε*_0_ the permittivity, *L* the length of the streaming channel and *A* the cross-section of the streaming channel. The surface zeta potential in the physiological pH range, the isoelectric point (IEP), the variation of zeta potential upon surface functionalization and coating, and the kinetics of protein absorption (serum) onto the MD surface can be evaluated by means of this approach [96,97].

### 5.6. Release of Nanomaterials from Medical Devices

The risk related to the use of nanostructures-based MDs is mainly associated with the possibility of nanoparticle release from the MDs, and their potential toxic effects. Nanomaterials could have a wide range of uses in MDs, as free nanomaterials (e.g., nano-silver in dressings), coatings (fixed onto a surface) or when loaded in the matrix structure (embedded) [89].

The release of nanoparticles could occur when they are released from a MD upon the matrix degradation or from the coated surface or could also originate from MDs degradation due to hydrolysis or catalysis. Invasive MDs could cause systemic exposure to nanoparticles and nanotoxicology should be carefully evaluated [89].

However, although the nanostructures are firmly part of the MD structure, their potential toxicity should be assessed since their peculiar physico–chemical properties increase their reactivity. Therefore, nanostructure identification and characterization are essential, including chemical composition, particle size and size distribution, shape and morphology, concentration and mass of particles, the surface charge. The evaluation of the nanomaterial release from a MD is challenging and currently a robust methodology to assess low level nanomaterial release is lacking.

If the MD is designed to be applied onto intact skin, the characterization of the release properties should take into account the dermal penetration of nanostructures and from this perspective an *in vitro* test using a Franz cell allows the assessment of the fraction of nanostructures released that are able to be absorbed.

### 5.7. Apyrogenicity and Sterility

Apyrogenicity and sterility are mandatory requirements for implantable MDs such as skin substitutes [12,90]. Traditional sterilization techniques are often unsuitable: gamma radiation is inappropriate for silk fibroin while steam sterilization modifies the PCL properties, and ethylene oxide sterilization could determine the presence of toxic residues. Sterilization could cause MD brittleness with a reduction in mechanical strength and elasticity [98]. For some materials, the application of classic sterilization protocols could cause a loss of crosslinking degree and the consequent change of chemico-physical properties, the production of free radicals or a change in particle size, significantly affecting safety, efficacy and system quality. Although there are ASTM (American Society for Testing and Materials) standards for the sterilization of collagen, chitosan and hyaluronic acid, for many materials it remains to investigate which is the most suitable method to ensure the sterility assurance level (SAL) [12,98].

Pyrogenicity is generally evaluated using the tests reported in the European and United States Pharmacopoeias, although the assays reported have been developed for traditional formulations.

Two different approaches are present: an *in vitro*/quantitative assay—the LAL (*Limulus polyphemus* amebocyte lysate) test and the *in vivo* test on rabbits. The LAL test is the preferred test and it is based on the capability of LAL to interact with pyrogens, and different methods are available: chromogenic (based on enzymatic reaction between pyrogens and LAL), turbidimetric (based on precipitation of endotoxin and LAL) or gel-clot ones (based on the clotting of LAL in presence of pyrogens). This allows the correlation of the pyrogen concentration to the response and the estimation of the pyrogen levels capable of inducing the release of proinflammatory cytokines and the severe response of the host [99].

Bacterial endotoxins, mainly lipopolysaccharides (LPS) from Gram-negative bacteria [100] are the more common pyrogens and the high surface area of nanostructures favors their adsorption, such as gold nanocrystals that inhibit the LAL response.

For this reason, pyrogenicity should be assayed *in vivo* on rabbits by the administration of the injectable into the animal and by monitoring an eventual body temperature increase caused by pyrogens [101].

## 6. Preclinical Evaluation of Medical Devices

Table 4 summaries Biological evaluation test of MDs (C: cytotoxicity; S: Sensitization; I/IR: Irritation/intracutaneous reactivity; S/ST: Systemic toxicity; G: Genotoxicity; I: Implantation; H: Hemocompatibility; CT: Chronic toxicity; CG: carcinogenicity; R/DT: Reproductive/developmental toxicity).

Preclinical evaluation is fundamental to rationalize the clinical trials and *in vitro* and *in vivo* approaches could furnish complementary results. In general, preclinical studies should be performed using GLP (good laboratory practice) and samples as similar as possible to the final product should be considered [102].

*In vitro* evaluation allows the evaluation of the MDs interaction with different cell lines, to assess cytotoxicity, hemolysis, cell uptake and platelet aggregation, and with proteins, such as those of the complemented system, and, in addition, in specific cases, the effectiveness on cell proliferation. *In vivo* evaluation allows the evaluation of the more complex aspects, including the effectiveness and toxicology for clinical translation.

Preclinical evaluation tests include cytotoxicity, sensitization, irritation, acute systemic toxicity, subacute/subchronic toxicity, genotoxicity, implantation, and hemocompatibility. Supplementary evaluation tests are chronic toxicity, carcinogenicity, reproductive/developmental toxicity and biodegradation. As shown in Table 4 from ISO 10993-1:2009 guidelines and described in the following paragraphs, cytotoxicity, sensitization and irritation (or intracutaneous reactivity) are mandatory for all MDs. Then, depending on the possible systemic distribution, the evaluation opens or closes further investigations, which include for example, genotoxicity and carcinogenicity. The more invasive and longer the contact of the device, the more toxicological endpoints should be considered.

### 6.1. Biocompatibility Testing

Cytotoxicity (C, Table 4) is a crucial aspect for an initial safety check. This is evaluated *in vitro* following protocols described in the ISO 10993-5 standard updated in 2009 [103]. The proposed scheme allows the specific evaluation of cell damage, the effect on cell growth, and specific aspects of cellular metabolism altered due to direct or indirect exposure to MDs. Normally the cytotoxicity test is carried out on cell cultures in monolayer. Different experimental conditions are suggested: (1) test by direct contact, in which the material is placed directly on the cell layer; (2) test on extracts, in which the sample is kept for 24 h at 37 °C in culture medium and the extraction liquid is then added to the cell layer; and (3) test by indirect contact, which includes filter diffusion and agar diffusion.

The MTT (3-(4,5-dimethylthiazol-2-Yl)-2,5-diphenyltetrazoliumbromide) assay is the most widespread one and it is based on the estimation of the cell mitochondrial activity after contact with the system compared to a control: NAD(P)H-(reduced nicotinamide adenine dinucleotide phosphate) dependent cellular oxidoreductase enzymes, such as mitochondrial dehydrogenases, are capable of reducing the tetrazolium dye (yellow color) to its insoluble formazan (purple color). If the cells remain viably in contact with the system, when MTT is added, mitochondrial dehydrogenases cause the opening of the tetrazolium ring with the formation of formazan salts, purple in color, which are dissolved in isopropanol and quantified by spectrophotometry [26].

Other cytotoxicity assessment methods include Neutral red uptake (NRU) cytotoxicity test, colony formation cytotoxicity test and XTT (2,3-bis(2-methoxy-4-nitro-5-sulfophenyl)-5-([phenylamino]carbonyl)-2*H*-tetrazolium hydroxide) cytotoxicity test. In any case, cell cultures should be chosen so that the test is much more predictive of the *in vivo* application [103].

The sensitization tests (S, Table 4), reported in ISO 10993-10, evaluate the potential of MDs to cause a sensitizing effect commonly causing skin redness and swelling or an allergenic reaction [104]. Sensitization is an immune response caused by the exposure to chemicals and in case of repeated exposures an allergic skin reaction arises. Due to the biological complexity, *in vivo* tests are required. Generally, guinea pigs are selected, and it is possible to observe the sensitization onset and the immune response related to repeated and prolonged interaction with MDs. For this purpose, the Buehler test and the guinea pig maximization test are performed. The Buehler test is used for devices in contact with skin such as electrodes and surgical drapes and is based on three phases: (1) induction phase: part of the MD is placed directly into contact with the shaved skin of the guinea pigs; (2) recovery phase to allow a response in the guinea pigs, and (3) triggering application phase: the entire test sample is applied in a single application.

The guinea pig maximization test is more sensitive than the previous one and it is used to test materials recommended for many application sites other than the skin. It involves the repeated application of material extracts emulsified with saline solutions or oils [105,106].

An innovative *in vitro* alternative method is the local lymph node assay (LLNA), which is more specific, reproducible, capable of providing quantitative information; this is based on cellular proliferation in the lymph nodes draining the site of topical application [107].

According to ISO 10993-10, irritation (I/IR, Table 4) is defined as a local inflammatory response to single, repeated and continuous applications of the test material, without an immune mechanism. Typical symptoms of irritation are redness, swelling, warming, and pain. MD extracts, emulsified with saline fluids or in vegetable oil, or MDs themselves are intracutaneously or ocularly applied: the extracts are injected intracutaneously in multiple points of the shaved skin of albino rabbits, or instilled in the precorneal area, respectively. After 24, 48 and 72 h, the onset of redness and swelling could be observed. Skin corrosion and irritation are classified into 1 (corrosive), 2 (irritant) and 3 (mild irritant) [108]. For skin irritation, the Draize rabbit skin irritation test is the preferred method, and it is performed using albino rabbits. The test substance is applied to the animal back and signs of irritation are observed, starting from a test animal to check the absence of corrosivity and confirming the test results using another two test animals [109,110].

### 6.2. Alternative In Vitro Tests for Irritation and Sensitization

Currently, in the EU there are *in vitro* validated tests to replace animal testing for irritation and skin sensitization [111,112]. *In vitro* skin irritation tests are validated on 3D reconstructed epidermis such as EpiDerm™, SkinEthic™, Episkin™ and LabCyte EPI-MODEL24 SIT [113,114,115,116,117]. The irritating potential of the substances is assessed on 3D models of reconstructed human epidermis by means of a topical exposure of a test substance followed by a cell viability test [118]. Additionally in these tests, cell viability is measured by MTT assay; recent studies showed that the MTT assay had more advantages than the IL-1α endpoint in the evaluation of skin irritants [119,120].

The 3D reconstructs represent the biological reference model for the replacement of animal experimentation, as they are closest in morphology and functionality to human tissues *in vivo*. The presence of a morphologically organized tissue with different cell layers is essential to quantify the MD biological impact, thanks to the biological relevance of the 3D-model, higher than that of single-layer cell cultures. The presence of a multi-layered structure allows the topical application of the product at the same doses and with the same frequencies used *in vivo* and takes into account its possible penetration [118]. It is also possible to evaluate the absence of interference with the physiological homeostasis of the tissue, the absence of toxic effects related to the product and to estimate the systemic effects considering penetration/permeation tests.

For example, Casas et al. tested an EpiDerm™ reconstructed human skin model with eleven polymer-based MDs using lactic acid and heptanoic acid as irritants. The irritation potential was assessed by MTT test and by proinflammatory secretion, demonstrating that this reconstructed human epithelium was a suitable *in vitro* replacement for the assessment of the irritation potential of MDs [121].

Moreover, the growing knowledge of the main biological and physiological mechanisms underlying skin sensitization, poses numerous alternative *in vitro* sensitization tests, recently accepted by the OECD (Organisation for Economic Co-operation and Development) [122], to evaluate chemical sensitizers.

In particular, these include the Direct Peptide Reactivity Assay (DPRA), KeratinoSens™ and the Human Cell Line Activation Test (h-CLAT) based on Adverse Outcome Pathway (AOP). DPRA is an acellular chemical test, proposed to evaluate the formation of a covalent bond between the allergen and the skin proteins, the initial molecular event of the skin sensitization process [123,124].

The KeratinoSens™ (Keratinocyte-based ARE-Nrf2 luciferase Reporter Gene Test Method) measures the activation of the cascade of molecular events under the control of the ARE element (Antioxidant/electrophile Response Element) in the HaCat cell line derived from human keratinocytes. Activation of this cascade controls the release of cytoprotective mediators in response to electrophilic and oxidative stress [125,126].

The h-CLAT test is based on the use of THP-1 cells (monocytic leukemia cell line), a pro-monocytic human cell line, used as a surrogate for dendritic cells to evaluate CD86 (a co-stimulatory molecule) and CD54 (an adhesion molecule) expression, associated with the monocyte and dendritic cell activation process [127]. These tests seem promising not only to replace sensitizing and irritating *in vivo* tests on MD but also on each single component.

### 6.3. Wound Healing Test

Wound healing test is considered as proof of concept for implantable MDs to treat chronic skin lesions. The basic principle of the *in vitro* wound healing test is the destruction of a cell monolayer, creating a cell-free area [128]. The basic information obtained from this assay is the scratch closure rate, and the migration rate to invade a free surface in a fixed time interval. The comparison between a prototype and a reference allows to obtain information related to system performance. For this purpose, microscopic investigations allow the verification of the gap closure advancement and cell morphology in a predetermined time interval. 

Different tests have been developed. The “scratch” test is the easiest wound healing test. A cell monolayer is etched with a pipette tip and the state of the cell culture, in the presence or in absence of the system, is observed at set times. However, the extent of the scratch depends on the researcher, as well as the identification of the wound margins being subjective, so the test presents numerous critical issues, mainly related to the poor reproducibility [129]. The stamp assay is based on the damage formation in a confluent cell substrate caused by a stamp mold strongly applied on it, manually or automatically generated. The mold is made of rubber [130] or poly(dimethyl)siloxane [131] or it can be characterized by specific patterns, inducing the formation of cellular debris as in a real wound. An important disadvantage is the irregularity of the printed area, especially if the pressure is applied without an automatic process [128]. The electric cell-substrate impedance sensing technique is based on the study of the impedance variation detected between two electrodes, proportional to cell division, adhesion and migration, as well as to the signal transduction and cytotoxicity. In this technique, an electrode is placed on the bottom of a plate, on which the cells grow, and another electrode is immersed in the culture medium. To avoid problems related to the application of too intense currents or the electrode deterioration, high frequency discharges (milliamps) are applied to the cells, in order to cause injury [132,133]. An alternative technique is the laser beam one to determine the insult in a confluent cell monolayer, offering high reproducibility of the lesion. The LEAP™ (laser enabled analysis and processing) instrument allows the creation of reproducible high-performance wounds in sterile conditions and uses only bright-field microscopy, avoiding problems related to the culture fluorescence staining [129].

The use of inserts shows evident positive effects on the assay, allowing the recreation in each sample of homogeneous and constant gaps thanks to their geometry. However, the cells could adhere to the insert and be torn from the monolayer when it is removed, leaving the edges indented. In addition, there could be a loss of adhesion of the insert from the bottom of the support (plate or well), allowing the cells to migrate early in the migration area. These conditions would cause a conformation of the gap flaps which is not uniform, having the same disadvantages of the handmade scratch [134].

**Figure 5:**
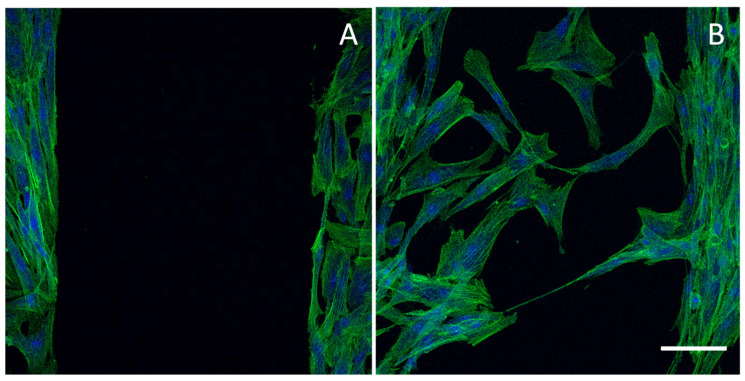
CLSM (confocal laser scanning microscope) images of a cell substrate (normal human dermal fibroblasts) with a free cell area (lesion) obtained using an insert (panel (**A**)) and the same substrate 24 h after the insert removal characterized by cells migrated/proliferated in the free area (panel (**B**)) (the cell substrate has been stained with phalloidin-FITC (green, F-actin filaments) and DAPI (blue, nucleus)). Scale bar 100 μm. Modified from [135]. CC BY 4.0.

Figure 5 reports CLSM (confocal laser scanning microscope) images of a cell substrate (normal human dermal fibroblasts) with a free cell area (lesion) obtained using an insert (panel A) and the same substrate 24 h after the insert removal characterized by cells migrated/proliferated in the free area (panel B) (the cell substrate has been stained with phalloidin-FITC (green, F-actin filaments) and DAPI (blue, nucleus)). 

### 6.4. Animal Wound Models

Animal injury models are used to evaluate the potential and/or adverse effects of MDs intended for wound treatment. Undoubtedly, for ethical and economic reasons, the philosophy of the 3Rs (reduction, replacement and refinement) should always be adopted in the conduction of the preclinical studies. Although research continues to improve *in vitro* tests, it is not possible to completely avoid animal testing. Not even the use of human tissue flaps *in vitro* allows the evaluation of systemic aspects that influence the wound healing, including for example endocrine interactions [136,137]. However, since no ideal model for chronic wounds or severe burns has been identified, animal models fairly fail to predict the outcome of clinical trials. For this reason, currently multiple animal models should be used to predict the MD ability of promoting wound healing. The pig model shows the greatest similarities with human skin, and it is recommended by FDA for the evaluation of re-epithelialization and wound contraction. Although the pig is recognized by the scientific world as the best model, the rodent is the most recurrent in the literature. Nevertheless, rodent skin anatomy differs from that of human due to their thin epidermis, dense hair, fast healing, absence of apocrine and eccrine glands and a better immune system [138,139,140].

Generally, dorsal incisions in rodents are the most common model. The lesions could be open or sutured before applying the formulation, depending on the clinical situation to be simulated. It is possible to recreate an ischemic gradient with the skin flap technique, which prevents blood circulation in a skin flap [136]. Hypoxia and re-epithelialization could be observed in dermal ulcers in the ears of rabbits, as well as the breaking force in a rat linear incision model [141]. The progress of the lesion status is generally monitored through photographs and histopathology of ulcer biopsies, aimed at establishing wound depth, state of proliferation and inflammation. Measurements of wound area are generally used to evaluate the initial and the final size of the wound, as well as the progress towards wound closure. Other parameters to be investigated are the wound index and the fall of the crust as an indicator of complete healing and the organization of ECM [142,143].

Due to the wide availability of wound models, as reported in Table 5, and the lack of standardization, it is often difficult to compare different studies.

Moreover, animal models are taken into account to study systemic toxicity, chronic toxicity, reproductive/developmental toxicity and carcinogenicity (ST, CT, R/DT, CG, Table 4), and the toxicity after implantation (I, Table 4).

## 7. Clinical Evaluation of Medical Devices

As for class III or implantable MDs, clinical investigations should be performed, unless existing clinical data are available and justifiable. Depending on the clinical indications and risk management outcomes, clinical investigations could also be necessary for non-implantable MDs [195].

**Figure 6:**
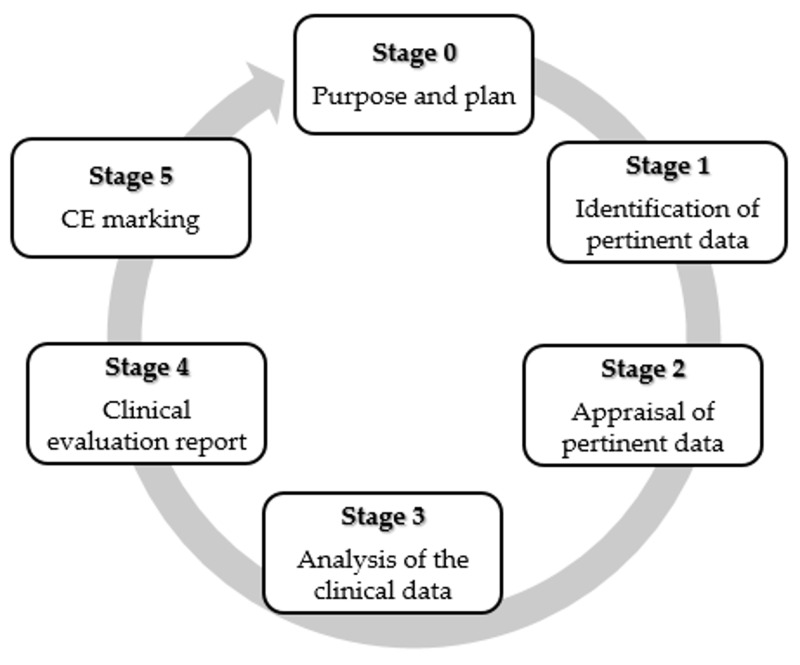
Different stages of the clinical evaluation. Modified from [195]. CC BY 4.0.

Since the preclinical tests, as previously described, do not allow the complete prediction of the performance of nanostructure based MD, clinical investigation is a mandatory requirement under current MD legislation to assess safety and the performance of nanotechnologies based MDs [196] The clinical trials of an MD can be defined as a process characterized by different stages, as shown in Figure 6. The manufacturer should develop a bibliographic research protocol that takes into account data sources, databases used, selection criteria applied and reasons for choice. The clinical investigation project should comply with relevant regulations, develop a careful risk management procedure and follow the appropriate ethical principles of the Helsinki Declaration, of the GCP (good clinical practice), of the UNI EN ISO 14155-1 (Clinical investigation of MDs for human subjects—General requirements) and UNI EN ISO 14155-2 (Clinical investigation of MDs for human subjects—Clinical investigation plans) standards [196]. Critical analysis based on relevant clinical and scientific data also in comparison to similar MDs should be performed. The authorization for clinical trials is provided by national and international agencies (such as the FDA or Ministry of Health) following the result verification of preclinical evaluations. The manufacturer notifies to the Ministry of Health at least 60 days before the scheduled start. After starting the clinical investigations, the manufacturer should communicate all serious adverse events (SAE) to the competent authorities involved [197,198].

Considering the difference among the various MDs it is difficult to assess a time to market, however on average, it takes about 18–24 months and typically the product design and development lasts 12–18 months (Stages 0–3), with an additional 3–6 months for regulatory approval (Stages 4–5).

## 8. CE Marking and Product Life Cycle

CE marking is fundamental to reach the market and the MD manufacturer should claim the product compliance with Medical Devices Regulation 2017/745, replacing Medical Devices Directive on 26 May 2020, and *In Vitro* Diagnostic Regulation 2017/746, replacing *In Vitro* Diagnostics Directive (IVDD) on 26 May 2022. Clinical trials could be performed on MDs not yet CE marked, or CE marked but substantially modified, or CE marked but not used for the intended use. These studies aim at CE marking and marketing (pre-market studies) or they could also be performed for research and study purposes (non-profit pre-market studies). Clinical trials could be also carried out on CE-marked MDs, as post-market studies to support product quality and post-market monitoring-surveillance plans. However, clinical trials with an MD not yet on the market are significantly riskier for the health of patients; therefore, these should be approved by both the Ministry of Health and the reference hospital Ethic Committees, where the investigation will take place. The hospitals involved should have specific competence and experience as assessed by the Ministry and the Ethics Committees [195,197,199]. In these cases, the safety and effectiveness of the device should be demonstrated. These data, together with those deriving from the scientific literature, the results of the pre-clinical tests, the data design and the specific technical documentation should be included in the clinical evaluation report (CER) that the manufacturer will submit to a Notified Body. The structure of a final study report is proposed in EN ISO 14155-1:2009. The CER should contain the general information necessary for the identification of the clinical investigation, the sponsor and the experimental centers, a summary of the clinical protocol, the description of the device under investigation, and the description of the statistical analysis plan of the results. Among others, the start and end date of the survey, the replacement of patients, the demographic characteristics of the study population should all be considered. Moreover, the compliance with the protocol and any deviations from the protocol should be reported. The analysis of the results obtained should include the adverse events and occurred effects, the device deficiencies and any corrective actions taken to resolve safety problems. The CER should be focused on a critical evaluation of the safety, performance and efficacy [198], the acceptability of the risk/benefit ratio and the risk assessment based on the use of the product on a large scale and long term [195,200]. If the evaluation is positive, the Notified Body issues the CE marking (with limited validity over a maximum time of 5 years). This certifies the safety and clinical performance of the device limited to the indications and procedures detailed in the protocols. In the post-market surveillance, the Notified Bodies confirm the CE marking.

**Figure 7:**
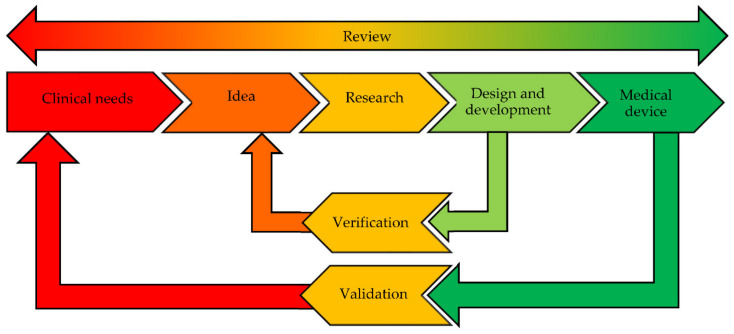
Design control documentation, evidence, and results in the life cycle for medical devices. Modified from [201] with permission.

The design controls cover the entire lifespan of the product: this does not end with the transfer of the design to manufacturing, but it rather applies to all changes and modifications to the device or to the manufacturing processes, including modifications that may occur after the device has been introduced to the market [201] (Figure 7).

Moreover, in the life cycle of a MD, a structured and periodic update, allows the manufacturer to demonstrate the clinical safety before and after the marketing phase [195,199].

## 9. Conclusions

Non-healing wounds generally have a negative impact on the patient quality of life and significantly increase the expenditure of the Healthcare System. Nanotechnology-based MDs should lead to potential clinical benefits and low complications, combining economic sustainability advantages, minimal preparation/storage and long shelf life (compared to the skin graft), making these products a promising treatment option for patients with chronic lesions. The low incidence of side effects supports acellular matrices as promising tools to enhance wound healing without immune response, compared to cell-based products.

However, the difficulties that nanotechnology-based MDs encounter along the path from research to the clinical practice are numerous. Relevant criticalities emerge from the purification of raw materials, as in the case of natural polymers, including also immunogenic potential. The application of conventional assays often leads to misleading results, and the analytical techniques are frequently incompatible with nanosized structures. Another critical issue is sterilization, especially when the matrices include biotechnological components. Furthermore, the preclinical *in vitro* and *in vivo* evaluations are particularly complex to recreate the wound microenvironment, both due to the wound heterogeneity and the lack of a universally recognized standard therapy. Animal models frequently involve species such as rodents, characterized by pathophysiological healing processes significantly different from those of humans. In addition to technical and scientific obstacles, the regulatory agencies are careful in considering the high innovation level and the unpredictable effects of nanotechnology-based MDs. A deep chemico–physical characterization and the identification of critical quality attributes (CQA) are mandatory for preclinical assessment and clinical evaluation.

In conclusion, although this is a complex scenario, great efforts from Regulatory Agencies and manufacturers are ongoing to promote nanotechnology-based MDs intended for the treatment of chronic wounds by harmonizing the characterization approach. This should have a beneficial impact for patients and the sector economy.

## Figures and Tables

**Table 1:** Risk classification and related examples of medical devices.

Class	Risk	Examples
I	Low	Examination gloves, colostomy bags or oxygen masks
IIa	Low/medium	Hearing aids, urinary and peripheral vascular catheters
IIb	Medium/high	Ventilators and intensive care monitoring equipment
III	High	Implants, balloon catheters, pacemakers

**Table 2:** Quality target product profile.

Intended use	Clinical treatment of skin chronic wounds
Device description	Nanotechnology-based medical device
Expected efficacy	Tissue reparation with minimal scarring
Quality	Control of the inter-individual response
Contraindications	No serious adverse effect
Preclinical testing	Cell adhesion and proliferation onto the systems
Clinical testing	Therapeutic efficacy

**Table 3:** Quality attributes (QA) for characterization of MDs.

QA		Description	Methods	References
**Physico-chemical chacterization**	Chemical and solid-state assessment	Information on chemical characterization of materials and surfaces, allowable limits for leachable substances, degradation products, tolerable intake for extractable substances, ethylene oxide sterilization residuals, physical form and crystal or amorphous form,	UV-vis, HPLC, GC/LC- MS, XRD, NMR; XPS	ISO 10993-7, 12, 13, 14, 15, 17, 18, 19
	Stability	Information on chemical, physical stability (tendency to aggregation, growth of crystals, variation of solubility and dissolution), chemical–physical stability related to the characteristics of the solid state such as morphology, as well as the adsorption of components of the formulation, assessment of the photostability and identification of storage conditions	FTIR, XRD, UV-vis, HPLC, GC/LC- MS	ICH Q1A (R2), Q1C (R2) and Q5C guidelines
	Particle size/morphology	Information on the primary and secondary particle size and system morphology	SEM, TEM, XRD, DLS, AFM, NTA, TRPS	ISO 10993-1
	Mechanical properties	Investigations on the biomechanical of the bioengineered matrix, and the properties that change when cells spread onto the system during its degradation.	Compressive, tensile and flexural tests	ISO 6892-1, ISO 178:2019
	Surface properties	Information on zeta potential of the medical device	LDV-PALS, Electrokinetic analyzer for solid surface analysis	ISO 10993-18
	Release of nanomaterials	Evaluation of the nanomaterial release from a medical device	Currently, a robust methodology, especially for the measurements of low level release of nanomaterials, is lacking.	ISO 10993-18
	Sterility and apirogenicity	Application of different methods of sterilization to investigate which is the most suitable method to ensure the SAL (sterility assurance level), and evaluation of apirogenicity	Steam and dry heat sterilization, ethylene oxide sterilization, LAL test	European and United State Ph.
**Biological characterizations**	Biocompatibility	Evaluation of cell damage, the effect on cell growth, and specific aspects of cellular metabolism altered following direct or indirect exposure with medical devices.	MTT assay, NRU cytotoxicity test, colony formation cytotoxicity test and XTT cytotoxicity test	ISO 10993-5
	Skin irritation	Evaluation of local inflammatory response to single, repeated and continuous applications of the test material, without an immune mechanism	Draize rabbit skin	ISO 10993-10
	Skin sensitization	Evaluation of the medical device potential to cause a sensitizing effect or an allergenic reaction	Buehler test, guinea pig maximization, LLNA assay	ISO 10993-10

**Table 4:** Biological evaluation test of MDs (C: cytotoxicity; S: Sensitization; I/IR: Irritation/intracutaneous reactivity; S/ST: Systemic toxicity; G: Genotoxicity; I: Implantation; H: Hemocompatibility; CT: Chronic toxicity; CG: carcinogenicity; R/DT: Reproductive/developmental toxicity).

Medical Devices			Biological Effect										
	Contact	Contact Duration	C	S	I/IR	ST	S/ST	G	I	H	CT	CG	R/DT
**Surface MD**	Skin	Limited	X	X	X								
		Prolonged	X	X	X								
		Permanent	X	X	X								
	Mucosal Membranes	Limited	X	X	X								
		Prolonged	X	X	X								
		Permanent	X	X	X		X	X			X		
	Breached/ compromised surface	Limited	X	X	X								
		Prolonged	X	X	X								
		Permanent	X	X	X		X	X			X		
**Ext. communicating MD**	Blood path indirect	Limited	X	X	X	X				X			
		Prolonged	X	X	X	X				X			
		Permanent	X	X		X	X	X		X	X	X	X
	Tissue, bone and dentin	Limited	X	X	X								
		Prolonged	X	X	X	X	X	X	X		X		
		Permanent	X	X	X	X	X	X	X		X	X	X
	Circulating blood	Limited	X	X	X	X				X			
		Prolonged	X	X	X	X	X	X	X	X	X		
		Permanent	X	X	X	X	X	X	X	X	X	X	X
**Implant MD**	Bone, tissue	Limited	X	X	X								
		Prolonged	X	X	X	X	X	X	X		X		
		Permanent	X	X	X	X	X	X	X		X	X	X
	Blood	Limited	X	X	X	X	X		X	X	X		
		Prolonged	X	X	X	X	X	X	X	X	X		
		Permanent	X	X	X	X	X	X	X	X	X	X	X

**Table 5:** Different types of wound models to study the wound healing process.

Model Injury	Animal	Method
Excisional wound	- Rodent [144,145,146,147] - Rabbit [148,149,150,151] - Pig [152,153]	Full-thickness circular excision
Incisional wound	- Rodent [143,154,155,156]	Linear/longitudinal incision
Burn wound	- Rodent [157,158,159] - Rabbit [160] - Pig [161,162]	Contact of skin with a heated metal [163,164], electricity [165], and heated water [163]
Burn/excisional wound	- Rodent [52,166,167] - Pig [168]	Contact of the skin with a hot device followed by full-thickness lesion
Excisional wound splinting	- Rodent [169,170,171]	Full-thickness excisional wounds followed by application of a splinting ring tightly around the wound, to inhibit wound skin contraction
Diabetic wound	- Rodent [172,173,174,175] - Rabbit [176,177,178] - Pig [179,180]	Transgenic db/db mice or induction of diabetes (Alloxan, Streptozotocin) followed by full thickness excisional wound
Infected Model	- Rodent [181,182,183] - Rabbit [184] - Pig [185,186]	Full-thickness wound followed by inoculation of bacteria (*P. aeruginosa*, *S. aureus*, *S. hyicus*)
Radiation-induced ulcer	- Rodent [187,188] - Rabbit [189] - Pig [190]	Radiation exposure followed by full-thickness excisional lesion
Dead space wound	- Rodent [191,192,193] - Pig [194]	Subcutaneous implantation of polypropylene tubes [109,110] below the skin
